# Phenotypic and genetic characterization of *vibrio cholerae* O1 isolated from various regions of Kenya between 2007 and 2010

**DOI:** 10.11604/pamj.2014.19.8.2496

**Published:** 2014-09-04

**Authors:** Njeru Mercy, Ahmed Abade Mohamed, Ng'ang'a Zipporah, Goutam Chowdhury, Gururaja Perumal Pazhani, Thandavarayan Ramamurthy, Hamadi I Boga, Samuel M Kariuki, Oundo Joseph

**Affiliations:** 1 Field Epidemiology and Laboratory Training Program, Kenya Ministry of Public Health and sanitation, Nairobi, Kenya; 2 Field Epidemiology and Laboratory Training Program, Tanzania; 3 Jomo Kenyatta University of Agriculture and Technology, Nairobi, Kenya; 4 National Institute for Cholera and Enteric Diseases (NICED), Kolkata, India; 5 Kenya Medical Research Institute, Kenya; 6 Centre for Disease Control (CDC), Nairobi, Kenya

**Keywords:** Cholera, characterization, Kenya, serotype, epidemics, pulsed-field gel electrophoresis

## Abstract

**Introduction:**

Cholera, a disease caused by Vibrio cholerae O1 and O139 remains an important public health problem globally. In the last decade, Kenya has experienced a steady increase of cholera cases. In 2009 alone, 11,769 cases were reported to the Ministry of Public Health and Sanitation. This study sought to describe the phenotypic characteristics of the isolated V. cholerae isolates.

**Methods:**

This was a laboratory based cross-sectional study that involved isolates from different cholera outbreaks. Seventy six Vibrio cholerae O1 strains from different geographical areas were used to represent 2007 to 2010 cholera epidemics in Kenya, and were characterized by serotyping, biotyping, polymerase chain r(PCR), pulsed-field gel electrophoresis (PFGE) and ribotyping along with antimicrobial susceptibility testing.

**Results:**

Seventy six Vibrio cholerae O1 strains from different geographical areas were used to represent 2007 to 2010 cholera epidemics in Kenya. Serotype Inaba was dominant (88.2%) compared to Ogawa. The isolates showed varying levels of antibiotic resistance ranging from 100% susceptible to tetracycline, doxycycline, ofloxacin, azithromycin, norfloxacin and ceftriaxone to 100% resistant to furazolidone, trimethoprim-sulfamethoxazole, polymyxin-B and streptomycin. The isolates were positive for ctxA, tcpA (El Tor), rtxC genes and were biotype El Tor variant harboring classical ctxB gene. All the isolates were classified as cholera toxin (CT) genotype 1 as they had mutation in the ctxB at positions 39 and 68. All the isolates had genetically similar NotI PFGE and BglI ribotype patterns. The absence of any observed variation is consistent with a clonal origin for all of the isolates.

**Conclusion:**

Kenya experienced cholera numerous outbreak from 2007-2010. The clinical Vibrio cholerae O1 isolates from the recent cholera epidemic were serotypes Inaba and Ogawa, Inaba being the predominant serotype. The Vibrio cholerae O1 strains were biotype El Tor variants that produce cholera toxin B (ctx B) of the classical type and were positive for ctxA, tcpA El Tor and rtxC genes.

## Introduction

Cholera is a widespread, severe diarrheal disease which continues to be a global threat [1]. The life-threatening diarrhea associated with cholera is attributed to massive luminal secretion of electrolytes and water from enterocytes, with elevated cyclic AMP levels induced by the cholera toxin (CT) produced by toxigenic Vibrio cholerae [2]. Since 1971, Kenya has suffered with many outbreaks of cholera. From 1974 to 1989, outbreaks were reported every year with an average case fatality rate of 3.6% [3]. Between 1997 and 1999, more than 33,400 notified cases of cholera were reported in Kenya, representing 10% of all *V. cholerae* cases reported from the African continent during this period [4, 5]. More cases have been reported locally since 2005, and the outbreak in 2007 had a case fatality of up to 5.6% [3, 6] which is over and above what is expected when proper intervention is instituted. There has been a steady increase of cholera cases in Kenya with 11,769 cases reported to Division of Disease Surveillance and Response (DDSR) of the Ministry of Public Health and Sanitation in 2009 (CFR = 2.3%) as compared with 3091 cases (CFR 3.7%) in 2008 [3]. This study sought to describe the phenotypic characteristics of the isolated *V. cholerae* isolates by serotyping, biotyping, detection of virulence genes, antimicrobial susceptibility testing, ribotyping and pulsed-field gel electrophoresis (PFGE).

## Methods

### Sources of *V.cholerae* isolates

Seventy-six *V.cholerae* isolates were identified from different geographic locations during 2007-2010 representing multiple cholera outbreaks. Of the 76 isolates, 56 were from multiple outbreaks at distinct geographic locations during 2009 and the other 20 isolates were from 2007, 2008 and 2010. The V. cholerae isolates were preserved at -80°C and revived by inoculation into alkaline peptone water at 37°C for 6 hrs and then subcultured on thiosulfate citrate bile salts sucrose agar (TCBS, EiKen Tokyo, Japan) and incubated at 37°C for 18-24 hrs. Suspected V. cholerae colonies were subcultured onto Muller-Hinton agar (Becton, Dickson (BD) and company, Sparks, MD, USA) plates and incubated at 37°C for 18-24 hrs [7]. All the isolates were confirmed by biochemical reactions based on sugar fermentation, oxidase test and serology using polyvalent O1, and monovalent Ogawa, and Inaba antisera. Of the 76 V. cholerae isolates, 44 representative ones were randomly selected for antimicrobial susceptibility testing and PFGE using NotI, based on their period of isolation and the area of the outbreak (3 isolates from 2007, 4 from 2008, 31 from 2009 and 6 from 2010). For ribotyping by BglI digestion, 26 strains were selected to represent cholera cases from various affected areas at different periods.

### Antimicrobial susceptibility testing

Antimicrobial susceptibility test was performed using commercial discs following manufacturer′s instructions (BD). Susceptibility to β-lactam antibiotics was tested using ampicillin (10 µg); susceptibility to cephalosporin's was determined using ceftriaxone (30ug). Ciprofloxacin (5 µg), norfloxacin (10 µg), ofloxacin and nalidixic acid (30 µg) were used for testing susceptibility to the quinolones. Aminoglycoside used in susceptibility tests was streptomycin (30 µg). Tetracycline antibiotics included doxycycline (30 µg) and tetracycline (30 µg). Other antibiotics included chloramphenicol (30 µg), furazolidone (100 µg), sulphamethoxazole (23.75 µg)-trimethoprim (1.25 µg) and azithromycin (15 µg). The inhibition zone diameters were recorded and the isolates were classified as susceptible, reduced susceptible or resistant to a particular antimicrobial agent using the Clinical and Laboratory Standards Institute guidelines [8]. Escherichia coli strain ATCC 25922 was used for quality control. The strains were tested for polymyxin B and sulphamethoxazole-trimethoprim minimum-inhibitory concentration using E-test strips (AB bioMeriuex, Solna, Sweden). *V. cholerae* strains O395 and N16961 were used as classical and El Tor biotype controls.

### PCR amplification

DNA used as template in the PCR reactions was prepared from overnight Luria-broth (BD) cultures by boiling for 10 min. PCR was carried out in 30 µl reaction volumes containing 3 µl 10X PCR buffer 2 µl of each of primer, 2 µl dNTPs mix (2.5 mM each dNTP, Roche, Mannheim, Germany), 0.2 µl (5,000 U/ml) Taq DNA polymerase (Roche), 3 µl of template DNA and 9.8 µl sterilized distilled water. Primers used in this study and the expected amplicon sizes are listed inTable 1. All PCR assays were performed using an automated thermal cycler (Gene Amp PCR System 9700; Applied Biosystems). PCR products were analyzed by electrophoresis in 1% agarose gels, stained with ethidium bromide, visualized under UV light, and recorded in a gel documentation system (Bio-Rad Laboratories, Hercules, CA, USA).

**Table 1:** List of PCR primers and aplicon sizes of target genes

Primers	Nucleotide sequence(5’-3’)	Amplicon size (bp)	References
ctxB (Fw-con)	ACTATCTTCAGCATATGCACATGG	947	Morita et al. (2008)
ctxB (Rv-classical)	CCTGGTACTTCTACTTGAAACG		
ctxB (Rv-El-Tor)	CCTGGTACTTCTACTTGAAACA		
tcpA, El Tor-Fw	GAAGAAGTTTGTAAAAGAAGAACAC	472	Keasler et al. (1993)
tcpA, El Tor-Rv	GAAGGAACCTTCTTTCACGTTG		
tcpA, classical-Fw	CACGATAAGAAAACCGGTCAAGAG	618	Keasler et al. (1993)
tcpA, classical-Rv	ACCAAATGCAACGCCGAATGGAGC		
rtxC-Fw	CGACGAAGATCATTGACGAC	265	Chow et al. (2001)
rtxC-Rv	CATCGTCGTTATGTGGTCGC		
CtxA-Fw	CTCAGACGGGATTTGTTAGGCACG	302	Keasler et al. (1993)
ctxA-Rv	TCTATCTCTGTAGCCCCTATTACG		
ctxB-F	GGTTGCTTCTCATCATCGAACCAC	400	Olsvik et al. (1993)
Ctxb-R	GATACACATAATAGAATTAAGGAT		

### Analysis of *V. cholerae* virulence genes

Using a multiplex/simplex PCR, all the isolates were screened for the presence of virulence encoding genes which included subunits of ctxA and ctxB (responsible for expression of of CT, tcpA (responsible for the expression of toxin-coregulated pilus) which is specific for El Tor and classical biotypes and rtxC (repeat in toxin) [9, 10] sequence difference at position 203 of the ctxB gene for the identification of CT of classical and El Tor biotype was done for all the 76 isolates. A conserved forward primer (FW-Com) and two allele-specific primers, Rv-cla and Rv-elt amplify ctxB of classical and El Tor biotypes respectively (Table 1) [11]. In addition, the ctxB gene was fully sequenced to determine additional mutations using the primers ctxB-F and ctxB-R.

### Ribotyping

Ribotyping was carried out with BglI digested genomic DNA. The digested DNA was separated in 1% agarose gels, transferred to nylon membrane (Hybond N + ; Amersham Pharmacia Biotech, Buckingham,UK) and hybridized with the alkaline phosphatase enzyme labeled 7.5-kb BamHI fragment of plasmid pKK 3535 [12] containing the 16S and 23S rRNA genes of Escherichia coli was used as the rRNA probe. The hybridized fragments were detected using a non-radioactive labeling kit using the substrate CDP-Star (Chemiluminescent Signal Detection and Generation, GE Healthcare, Amersham Pharmacia Biotech, Buckingham, UK) following the procedures described by the manufacturer. The molecular marker was a 1kb DNA ladder (Takara, Shiga, Japan).

### PFGE

PFGE was performed using the PulseNet recommended procedure. [13] The plugs containing agarose embedded V. cholerae DNA were digested with 50 Units/µl of NotI (Takara). For digestion of Salmonella Braenderup H9812 the molecular size marker, XbaI (15 Units/µl) was used and the DNA fragments were separated electrophoretically under the contour-clamped homogeneous electric field (CHEF) on a CHEF DRIII system (Bio-Rad) on 1% PFGE grade agarose gel in 0.5xTBE (44.5 mM Tris/HCl, 44.5 mM boric acid, 1.0 mM EDTA, pH 8.0) at 14°C. Following electrophoresis, the gels were stained for 30 min in Elix MilliQ water (Millipore, Bangalore, India) containing 1.0 µg ethidium bromide, destained in Elix MilliQ water for 15 min and photographed under UV light using the Gel Doc 2000 gel documentation system (Bio-Rad). DNA fingerprint patterns were analyzed using a computer software package, Bionumeric (Applied Maths, Belgium). Fingerprint patterns were typed on the basis of banding similarity using Dice similarity coefficient and unweighted-pair group method using average linkages (UPGMA) clustering methods.

### Ethical consideration

Approval to carry out this study was obtained from the Ministry of Public Health and Sanitation and Kenya Medical Research Institute (KEMRI) Scientific Steering Committee (SSC) and National Ethical Review Committee (ERC), through Centre for Microbiology Research (CMR). Authority to use archived isolates under the custody of National Public Health Laboratories mandated by the Ministry of Public Health and Sanitation to deal with outbreak samples was obtained.

## Results

**Antimicrobial susceptibility testing**: All the 44 isolates were susceptible to tetracycline, doxycycline, ofloxacin, azithromycin, norfloxacin and ceftriaxone, and resistant to furazolidone, streptomycin and sulfamethoxazole-trimethoprim. Thirty-nine (89%) isolates were resistant to nalidixic acid, 18 (41%) to ampicillin, and three (7%) to chloramphenicol. Reduced susceptibility to ciprofloxacin, nalidixic acid, ampicillin and chloramphenicol was observed in 2.3%, 59.1%, 11.4%, and 93.2% of the isolates, respectively.

**Biotype specific traits**: All of the isolates were determined to have the El Tor biotype. The minimum inhibitory concentration (MIC) using polymyxin B test strip showed that eighty percent (80%) of the strains had a MIC of 128µg/ml and others (20%) had a MIC of 192µg/ml as did the El Tor (N16961) control. The classical (0395) control strain had an MIC of 1.5µg/ml. The multiplex and simplex PCR assays showed that all the Kenyan V. cholerae isolates were positive for ctxA, tcpA (El Tor) and rtxC genes, as expected for the El Tor biotype. The MAMA-PCR assay identified all the isolates as V. cholerae O1 El Tor variant strains since they harbored the classical ctxB gene. In addition, the ctxB sequence had the known variants at positions 20, 39, 68.

**Figure 1:**
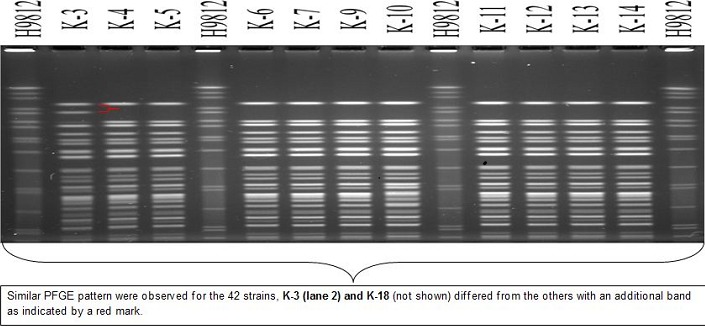
PFGE of Not 1 digested genomic DNA of V. Cholera O1 Inaba and Ogawa strains isolated from various regions of Kenya between 2007 and 2010. H9812: Molecular weight marker (S. Braenderup digested with Xba1 restriction enzyme), K-3 (Namanga 2010), K-4 (Kisumu 2009), K-5 (Migori 2008), K-6 (Migori 2008), K-7 (Moyale 2009), K-9 (Mukuru 2009),K-10 (Mbagathi 2009), K-11 (Dadaab 2007), K-12 (Namanga 2010), K-13 (Kisumu 2008), K-14 (Manyatta Kisumu 2009)

**Figure 2:**
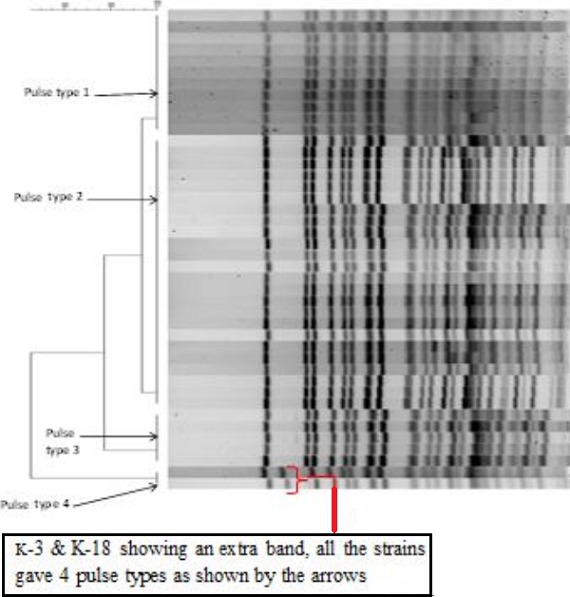
Dendogram of V. cholera O1 strains from different geographical regions, Kenya, 2007-2010

**PFGE and ribotyping results of Bgl I digested chromosomal DNA**: The genomic DNA from the 44 representative isolates digested with Not1 restriction enzyme gave similar PFGE patterns irrespective of year and place of isolation (Figure 1). Strains K-3 (Namanga 2010) and K-18 (Westpokot 2010) had one extra band. All the tested isolates belonged to ribotype RIII (data not shown). When analyzed with the Bionumerics software 4.0 package, all the isolates gave 4 pulsotypes. Isolates belongs to these pulsotypes were 100% similar within the group. Overall, all the isolates exhibited an average similarity of 95.5% irrespective of place and year of isolation (Figure 2)

## Discussion

In this study, a total of 76 isolates were biochemically identified as V. cholerae and serologically confirmed as serogroup O1, serotype Inaba/Ogawa. Cholera outbreaks in different provinces of Kenya in 1970s through 1990s and during 1998-99, were mainly caused by the serotype Ogawa [14, 15]. Our recent observation indicates the predominance of serotype Inaba. The serotype switchover from O1 Ogawa to Inaba and vice versa is common in many cholera endemic areas [16]. Kenyan V. cholerae isolates were resistant for chloramphenicol, streptomycin, sulphonamides, sulfamethoxazole and trimethoprim for isolates from 2007 to 2010 [17]. PCR analysis revealed all the isolate were positive for ctxB of classical type, rtxC, tcpA (El Tor) and ctxA genes. There are two biotypes of Vibrio cholerae O1 serogroup which are El Tor and classical. There are three variants of El Tor biotypes of V. cholerae O1 that have been described, which include; The Matlab variants, which cannot be biotyped, having a combination of classical and El Tor features, [18, 19], the Mozambique variants that have a typical El Tor genome but a tandem repeat of classical prophage located in the small chromosome, [20] and the altered El Tor strains, which have an El Tor CTX prophage but produce CT of the classical type [21]. The MAMA-PCR assay for the differentiation of the CTB subunit of classical and El Tor biotypes of V. cholerae indicated that all the 76 isolates were El Tor variant that harbors the classical ctxB gene. The strains showed properties similar to the El Tor biotype such as presence of gene encoding tcpA (El Tor) and resistance to polymyxin B. It is thought that El Tor strains have greater adaptability and survival capacity in the environment, colonize better in the intestinal epithelium and have a more efficient host-to-host transmission than classical strains [22]. In addition, El Tor variants able to produce the CT similar to that of classical biotype that enhances the virulence. [23]. Combination of these two features may have supported the El Tor variant to survive and spread the disease with high morbidity and mortality as seen in recent years. Findings of this study are similar to recent reports, which have shown the spread of El Tor strains harboring classical CT gene in several countries in Asia, Africa and Haiti [24, 25]. V. cholerae O1 strains isolated from Orissa in eastern India during 2007, Nigeria and Cameroon in 2009 and Haiti in 2010 had three mutations in the CtxB at positions 20 (replacement of histidine by asparagine), 39 (histidine in place of tyrosine) and 68 (threonine in place of isoleucine). However, all our tested isolates had two variant nucleotides at positions 39 and 68, indicating that they are clonally different from West African and Haiti isolates.

Antimicrobials are known to be effective against *V.cholerae* to shorten the duration of illness and reduce volume of stools. WHO recommends that cholera patients be treated with tetracycline, doxycycline, furazolidone, sulphamethoxazole-trimethoprim, chloramphenicol or ciprofloxacin. However in this study, the isolates were resistant to furazolidone, streptomycin and sulfamethoxazole-trimethoprim. Thus, two of the six WHO recommended antibiotics are unlikely to be effective for cholera patients in Kenya. Previous studies show that *V.cholerae* O1 strains isolated in Kenya during 1982-1985 were resistant to tetracycline, ampicillin and sulfamethoxazole-trimethoprim [26]. These findings were similar to the one recorded during 1998-1999 cholera epidemic in Kenya [14]. All the isolates in this study were resistant to furazolidone which differs with the findings from Kenya during 1994-2007 where none were resistant to furazolidone [26]. Multiple antibiotic resistances among V. cholerae has emerged as a major problem worldwide [27]. *V.cholerae* O1 strains isolated between 1994 and 1996 from six countries from East Africa region (Kenya, Sudan, Somali, Tanzania, Rwanda and Mogadishu) exhibited no uniformity in the resistant pattern.. The main observation was that the strains from Kenya and Sudan were susceptible to tetracycline [28]. In a previous study using strains from outbreaks in 2005, 97% were susceptible to tetracycline [6]. Isolates used in this study were susceptible to tetracycline and doxycycline which are the drugs currently used in treatment of cholera cases in Kenya. This is different from reports from other regions like India showing a re-emergence of tetracycline resistant strains [29]. All the isolates were susceptible to azithromycin that can be used as a substitute drug if resistance for other potent antimicrobials continues. All the V. cholerae O1 isolates showed high levels of resistance (32.0µg/ml) to trimethoprim-sulfamethoxazole, which raise concerns especially because this drug is increasingly being used as prophylaxis for HIV-opportunistic infections [30]. In this study, the isolates were susceptible to ciprofloxacin, ofloxacin, and norfloxacin but resistant to nalidixic acid (88.6%)-a non-fluorinated quinolone. Reduced susceptibility to ciprofloxacin has been reported from Zimbabwe, Nigeria and Cameroon [31, 32]

Ribotyping has been successfully used to demonstrate the emergence and progression of different clones of V. cholerae strainsin Bangladesh [33]. It has been reported that V. cholerae O1 that were isolated before the O139 outbreak in late 1992 had a typical Bgl I ribotype profile named RI. The RII and RIII Bgl I ribotype profiles were assigned to V. cholerae O1 strains isolated during and after the O139 outbreaks [34]. All the 2007 to 2010 Kenyan O1 isolates displayed RIII ribotype, which would have spread from regions where O139 was dominant and spread to Kenya after 1995. In the PFGE, Not1 digested genomic DNA of V. cholerae O1 Inaba and Ogawa isolates produced 4 pulsotypes except for two isolates (K-3 from Namanga, K-18 from West Pokot). These two isolates were from the 2010 cholera outbreaks in Rift Valley province, Kenya. In the dendrogram, the overall similarity of 44 isolates was 95%. This strongly suggests that V. cholerae O1 that caused outbreaks from 2007 to May 2010 are clonally related. It appears that multiple foci of genetically similar strains of V. cholerae O1 that exist in the local environmental reservoirs. Clonal distribution of V. cholerae have been seen in many parts of Kenya. [35, 36] Isolates from 5 outbreaks between January and June 2005 were V. cholerae O1, Inaba serotype, and had genetically similar in the SfiI PFGE patterns. [6] A study on V. cholerae O1 isolates causing outbreaks in Kenya between 1994 and 2007 showed identical patterns in antibiotic susceptibility, virulence genes, Not1 PFGE and the diversity in the mobile genetic elements, suggesting that the O1 strains associated those outbreaks were clonally related [26].

A recent publication concluded that V. cholerae isolates from Kenya, including many reported on here, were not clonally related [37]. The conclusion was based on multilocus variable tandem repeat analysis which showed that that isolates from 2009 and 2010 were highly variable in contrast to the genetic and phenotypic uniformity in our data. The presence of some isolates Fin both studies confirms that PFGE, ribotyping, ctx typing and antibiotic resistance typing are all detect less variation than tandem repeat analsyis and thus although the data presented in this paper properly conclude that *V. cholerae* isolates in Kenya are clonal, it does not contradict the conclusion from tandem repeat analysis that the Kenyan isolates are not clonal.

## Conclusion

*V. cholerae* O1 isolates collected between 2007 and 2010 from different parts of Kenya had similar antimicrobial susceptibility patterns, toxin and virulence genes, ribotype and PFGE patterns. Our data reveal that the isolates are resistant to furazolidone, streptomycin and sulfamethoxazole-trimethoprim and hence these antibiotics should not be used for treatment of cholera in Kenya, However, the isolates were sensitive to tetracycline and doxycycline that are currently used in treatment of cholerae cases in Kenya and thus should be continued to be in the treatment of cholerae cases.
